# Superpixel Segmentation Based Synthetic Classifications with Clear Boundary Information for a Legged Robot

**DOI:** 10.3390/s18092808

**Published:** 2018-08-25

**Authors:** Yaguang Zhu, Kailu Luo, Chao Ma, Qiong Liu, Bo Jin

**Affiliations:** 1 Key Laboratory of Road Construction Technology and Equipment of MOE, Chang’an University, Xi’an 710064, China; luokailu@chd.edu.cn (K.L.); machao@chd.edu.cn (C.M.); liuqiong@chd.edu.cn (Q.L.); 2 State Key Laboratory of Fluid Power & Mechatronic Systems, Zhejiang University, Hangzhou 310028, China; bjin@zju.edu.cn

**Keywords:** boundary information, legged robot, superpixel segmentation, terrain classification

## Abstract

In view of terrain classification of the autonomous multi-legged walking robots, two synthetic classification methods for terrain classification, Simple Linear Iterative Clustering based Support Vector Machine (SLIC-SVM) and Simple Linear Iterative Clustering based SegNet (SLIC-SegNet), are proposed. SLIC-SVM is proposed to solve the problem that the SVM can only output a single terrain label and fails to identify the mixed terrain. The SLIC-SegNet single-input multi-output terrain classification model is derived to improve the applicability of the terrain classifier. Since terrain classification results of high quality for legged robot use are hard to gain, the SLIC-SegNet obtains the satisfied information without too much effort. A series of experiments on regular terrain, irregular terrain and mixed terrain were conducted to present that both superpixel segmentation based synthetic classification methods can supply reliable mixed terrain classification result with clear boundary information and will put the terrain depending gait selection and path planning of the multi-legged robots into practice.

## 1. Introduction

A multi-legged robot that originates from bionic of reptiles has high walking stability and low energy consumption in a stationary state. Due to its redundant limb structure, it manages good stability even in the complex environment [1,2]. Compared with a wheeled robot, a multi-legged robot can cross large obstacles and has many degrees of freedom that contribute to better flexibility and adaptability, so that the legged robot has a wide range of application. The researchers have designed different multi-legged robots, such as mines sweeping robot [3], volcano detecting robot [4], underwater robot [5], strawberry picking robot [6] and other robot prototypes. As a multi-legged robot represents a nonlinear, multi-body, rigid–flexible system having the complex interactions with the environment, the environmental characteristics have a great influence on robot mobility. If a robot cannot accurately recognize the terrain, it may make a wrong gait plan. Therefore, correct perception and ability to classify the terrain are necessary to make the correct gait planning, path planning and motion control strategy in time. To ensure robot adaptability to the environment and its ability to independently choose the region, and avoid the problems in stability movement control such as slipping and instability in the process of motion, it is necessary to improve robot ability to perceive different terrain characteristics. During the interaction between the robot and environment, both geometric and non-geometric features of the terrain influence the robot’s performance. On single, flat terrain, the robot can use the periodic gait to walk quickly and smoothly. However, on complex terrain, the robot must use sensor information and an appropriate gait planning method to control its contact point and position in real time to improve its safety and efficiency. 

At present, sensor-based robot terrain classification methods are widely used. Compared with other sensors, visual sensors are the most similar to the human perception of the environment and can provide information from images. By extracting different information, such as spectrum [7], color [8] and the local features of the terrain [9], terrains can be accurately identified. The terrain classification method proposed by Zenker et al. [10] is a topographic surface classifier which extracts local features and color features. Filitchkin et al. [11] used a gradient descent heuristic algorithm to adjust the SURF Hessian threshold to achieve nominal feature density. These methods focused on improving the classification by using the accurate feature values. The existing deep convolution neural network classification and segmentation architectures are suitable for terrain segmentation and recognition. They can solve the problem that the SVM classification process is complex and needs multiple inputs to distinguish a mixed terrain. Thus, CNN-based terrain classification is suitable for a variety of mixed terrains. Bao et al. [12] proposed a novel method for brain MR image segmentation, with deep learning techniques to obtain preliminary labeling and graphical models to produce the final result. Rothrock et al. [13] completed the terrain classification of Mars rover missions based on deep learning. 

For a robot capable of autonomous navigation in a complex outdoor environment, the terrain classification process requires not only accurate terrain classification results, but also clear boundary information of different terrains. Therefore, image segmentation techniques are introduced here to complete the division and labeling of different terrain boundaries. The existing commonly used image segmentation methods mainly have the following methods. Turbopixel/superpixel segmentation methods [14,15,16,17]: Nguyen et al. [18] integrated the edge detector into the superpixel algorithm and customized the multi-channel image to improve the superpixel segmentation and used to segment mouse regenerative muscle fibers. Chen et al. [19] proposed a super-pixel based automatic brain tumor segmentation framework. Watershed segmentation methods: Marcin [20] outlined the watershed by immersion segmentation to identify the coronal hole regions in the solar image acquired using the Extreme Ultraviolet Imaging Telescope. Cousty et al. [21] introduced watershed cutting, the watershed concept in edge-weighted graphs, and derived a three-factor watershed cutting strategy through a sparse paradigm. Level-set techniques: Zhang et al. [22] performed image segmentation under intensity inhomogeneity. Min et al. [23] proposed a new level set segmentation model integrating the intensity and texture terms for segmenting complex two-phase nature images. The watershed image segmentation algorithm has the advantages of low calculation cost and high segmentation precision. The watershed transformation is performed by segmenting the morphological gradient signal of the image. Although level-set technique has strong anti-noise ability, it cannot solve the weak edge problem of complex images.

Although the use of terrain classification can obtain results with semantic filling, its boundary classification for mixed terrain is unclear, which will not make the robot be able to make correct gait transformation in time at different terrain boundaries. This will affect the stability requirements of the robot. Therefore, superpixel segmentation based synthetic classifications are proposed to make the robot be able to judge the terrain timely and perform appropriate gait transformation and path planning. Hence, environmental adaptability and self-selection capabilities of the robot during the movement are improved. Moreover, the terrain classification system is perfected, and the existing terrain classification methods are improved. Lastly, the robot ability to work autonomously outdoors is achieved.

The paper is organized as follows. In Section 2, the existing problems in terrain recognition by outdoor legged robots are presented. The SLIC-SVM and SLIC-SegNet terrain classification methods are proposed as a solution for these problems and are respectively introduced in Section 3. In Section 4, the experiments with the proposed terrain classification methods are presented, and the achieved classification results are provided and analyzed. The discussion of results is given in Section 5. Lastly, the conclusions are given in Section 6.

## 2. Motivations

**Figure 1:**
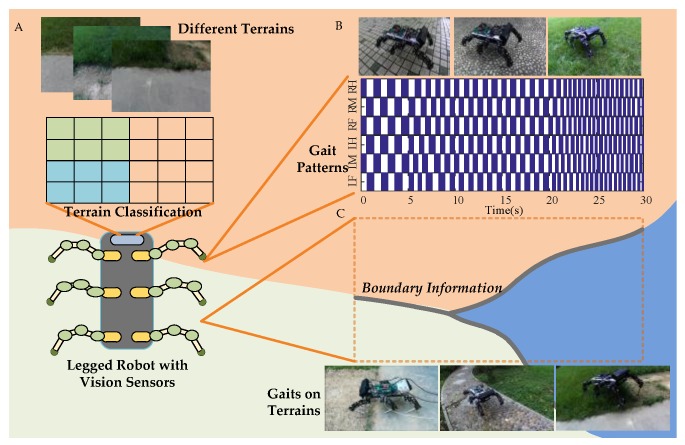
Hexapod robot and terrain classification. (**A**) stands for different terrains. (**B**) represents the gait of the robot in single terrains. (**C**) represents the gait in mixed terrains.

Nowadays, most of the control methods used in the multi-legged robots are highly-dependent on the environment and can provide real-time environmental information to the control system of the multi-legged robots, which is the basis for robotic decision-making. To improve the robot stability and adaptability to the environment, the multi-legged robot must have the ability for environment detection and classification in the complex unstructured environment. The robot’s perception and detection of the environment mainly depend on obtained visual information. Visual-based terrain recognition helps the robot to understand the upcoming terrain, make reasonable predictions, and classify terrain by appropriate terrain classification methods. The information on environment is fed back to the robot to select the most appropriate gait. The establishment of a reasonable terrain classifier and recognition of the surface texture features are crucial for robot terrain recognition. Currently, the most widely used classification methods are SVM [10,11,24], neural networks [25,26], deviation classifiers [27] and Gaussian mixture [28]. Most of them have good adaptability and high recognition accuracy and widely used in many occasions. However, since the multi-legged robots are mostly used in the complex, unstructured, tough environment, these terrain classifiers have some limitations and cannot meet the classification requirements for movement on the mixed terrain. The terrain classification process of multi-legged robots in complex unstructured outdoor environments mainly has the following concerns:

Terrain Classification. Most terrain classification methods have limited identifiable terrain samples and cannot be applied to complex and variable unstructured outdoor environments, resulting in terrain identification errors and unrecognized terrain types. In SVM [29] and Segnet [30], the recognition accuracy depends on the number of samples. A series of mixed terrains is presented in Figure 1A.

Gait Selection. A multi-legged robot on different terrains has different contact characteristics during interaction with the ground. The information on terrain characteristics includes geological characteristics for gait selection and estimation of reaction force, which has certain significance to prevent the robot from sliding. Robot stability is always evaluated by estimating the terrain properties [31] and various gaits effect on the movement behavior and different sensory patterns are also considered [32]. Different gait patterns of a multi-legged robot are presented in Figure 1B [33].

Boundary Information. The classification of terrain types and the recognition of terrain boundaries determine the gait used by the robot. The terrain classification process can be quickly realized by terrain classification methods, but always fail to gain clear boundary information, which leads the robot to change gaits in improper moment. Sometimes the balance of the robot will be affected. The influence of boundary information on gait selection under various mixed terrain is presented in Figure 1C.

## 3. Methods

Therefore, to solve the above problems, two terrain classification methods are proposed: SLIC-SVM and SLIC-SegNet. In SLIC-SVM, the mixed terrain is segmented into sub images by SLIC. The feature extraction in sub images is performed and the terrain is identified by SVM. A new terrain classification method, SLIC-SegNet, is also proposed. Here, the superpixel segmentation method is used to solve the problem of an unclear boundary of the SegNet recognition that leads to inaccurate gait transformation at the intersection of different terrains. Thus, the terrain classification with clear boundaries and fillers with meaning are obtained.

### 3.1. Superpixel Segmentation for Clear Boundary Information

In the image segmentation field, superpixel image preprocessing techniques have been rapidly developed in recent years. The concept of superpixels which quickly partition the image into multiple subregions with image semantics was first proposed by Ren et al. [15]. Compared with the traditional processing methods, the extraction and expression of superpixels are more conducive to the collection of image local features. The existing segmentation algorithms usually have a limit on the number of pixels, compactness, segmentation quality, and algorithm application. Song et al. [16] evaluated all the existing superpixel segmentation algorithms. Their results show that the SLIC superpixel segmentation algorithm has good performance in controllability and controllability of the number of superpixels. Due to the segmentation problem, the SLIC segmentation algorithm is applied to the mixed terrain, and most pixels are selected as target regions in a plurality of superpixel regions and the boundary pixels of the pixel coordinates of the fitted curve are extracted as the terrain boundary segmentation of the complex terrain image. The SLIC algorithm is performed as follows:

1. Initialize the cluster center. According to the set number of superpixels *K*, evenly distribute the seed points in the image. The superpixel size is *N*/*K*, where *N* is the number of pixels.
2. Calculate the gradient values of all pixels in the seed points’ neighborhood, and move the cluster center to the position of the lowest gradient within the n×n grid that contains the pixels to reduce the chance of selecting noisy pixels.
3. Assign a class label to each pixel in the neighborhood of each reselected cluster center. The search range is 2 *S* × 2 *S*. The desired superpixel size is *S* × *S*.
4. Distance metrics. The SLIC clustering is based on color similarity and proximity between pixels, where the measure of color similarity is (*l*, *a*, *b*), the color space norm, and the measure of color proximity is the two-dimensional coordinate space of the image (*x*, *y*). Therefore, the comprehensive metric factor is the five-dimensional space, [*l*, *a*, *b*, *x*, *y*]. For each pixel, its distance from the seed point is calculated. The corresponding distances are calculated by:
  (1) $$d_{c}=\sqrt{{\left(l_{j}-l_{i}\right)}^{2}+{\left(a_{j}-a_{i}\right)}^{2}+{\left(b_{j}-b_{i}\right)}^{2}},$$
  (2) $$d_{s}=\sqrt{{\left(x_{j}-x_{i}\right)}^{2}+{\left(y_{j}-y_{i}\right)}^{2}},$$
  (3) $$D^{\prime }=\sqrt{{\left(d_{c}/N_{c}\right)}^{2}+{\left(d_{s}/N_{s}\right)}^{2}},$$
  where *d_c_* represents the color distance, *d_s_* represents the spatial distance, *N_s_* is the maximum spatial distance in the cluster, and *N_s_* = *s* = *sqrt*(*N*/*K*). The maximum color distance *N_c_* varies from picture to picture and from cluster to cluster, here we take a fixed constant (value range [1, 40], generally 10). Since every pixel is searched by multiple seed points, every pixel has a certain distance from the surrounding seed points, and the seed point corresponding to the minimum value is used as the clustering center of a pixel.
5. Iterative optimization is performed by:
  (4) $$G(x,y)=\|I\left(x+1,y\right)-{I(x-1,y)\|}^{2}+\|I\left(x,y+1\right)-{I(x,y-1)\|}^{2},$$
  where *I* (*x*, *y*) denotes the experimental vector corresponding to the pixel position (*x*, *y*) and it denotes a norm. After each pixel in the image is associated with the cluster center, a new center is obtained as the average experimental vector, and each pixel is continuously and iteratively associated with the nearest cluster center, and the cluster center is recalculated until the process convergence is achieved.
6. Enhanced Connectivity. Distribute discontinuous superpixels and oversized superpixels to the neighboring superpixels. The traversed pixels are assigned to the corresponding labels until all points are traversed.

**Figure 2:**
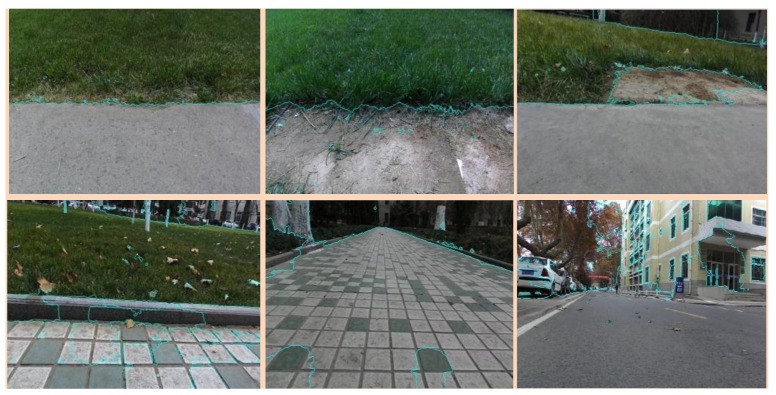
Superpixel segmentation results.

After superpixel segmentation, the boundaries of the mixed terrain are clearly divided. It provides a boundary basis for image segmentation in SVM mixed terrain classification and boundary segmentation of SegNet terrain classification results. This ensures the stability requirements of the robot at the boundary of the mixed terrain. Figure 2 shows examples of the superpixel segmentation. 

### 3.2. SLIC-SVM Terrain Classification 

**Figure 3:**
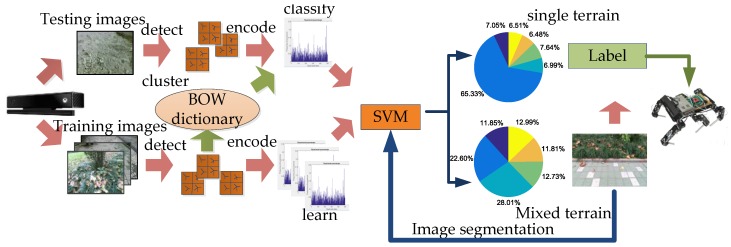
SLIC-SVM terrain classification system.

The SURF method is a commonly used local feature extraction in image classification. The SURF detector essentially relies on the Hessian matrix. To localize key points, SURF interpolates the local maxima of the determinant of the Hessian matrix in scale-space. Instead of gradients, a distribution of Haar-wavelet response is used [34]. Then, the Bag-of-words (BOW) model [35] is used to establish a terrain classifier that clusters the extracted feature points, which represent visual vocabulary in the work. Then, the terrain image is encoded to generate the visual vocabulary dictionary and visual vocabulary frequency histogram corresponding to each terrain type. Finally, the information is trained by SVM. The image labeled by terrain is further processed after the SVM classification and the confidence that the image belongs to various types of terrain is obtained. Determine the terrain type by confidence. If it is a single terrain, directly output the terrain label. If it is a mixed terrain, first use the SLIC to divide the boundary, and then repeat the SVM terrain classification process after image segmentation until the output is a single terrain label to complete the terrain. The terrain classification process is shown in Figure 3. 

In the SVM, the optimal hyperplane is divided between terrain types to classify various terrain types. The following is the construction method of the optimal hyperplane. Defining classification function *f*(*X*) = *ω^T^X + b*. When *f*(*X*) = 0, X is the point on the hyperplane. The sample point with *f*(*X*) > 0 corresponds to the data point with the label *Y* = 1. The sample point with *f*(*X*) < 0 corresponds to the point where the label is *Y* = −1. In the case where the hyperplane *ωX* + *b* = 0 is determined, |*ωX* + *b*| is used to represent the distance from any point *X* to the hyperplane. We can use the positive and negative of (*Y*(*ωX* + *b*)) to indicate the correctness of the classification.

Define function interval $\hat{\gamma }$: *f*(*X*_0_) = 0:

(5) $$\hat{\gamma }=Y(\omega^{T}X+b)=f(X).$$

Assume that the point *X* is vertically projected onto the hyperplane is *X*_0_, *ω* is the normal vector of the hyperplane, and *γ* is the length of *XX*_0_, which is the distance from the sample *X* to the hyperplane.

(6) $$X=X_{0}+\gamma \frac{\omega }{\|\omega \|,}$$

in which, ‖ω‖ is the second-order norm of *ω*. Satisfy *f*(*X*_0_) = 0, available *ω^T^X*_0_ = *b*. Substituting Equation (6), multiply both sides by $\omega^{T}$ to calculate:

(7) $$\gamma =\frac{\omega^{T}X+b}{\left\|\omega \right\|}=\frac{f(X)}{\left\|\omega \right\|}.$$

Then, the definition of the geometric interval (indicated by $\tilde{\gamma }$):

(8) $$\tilde{\gamma }=Y\gamma =\frac{\hat{\gamma }}{\left\|\omega \right\|}.$$

When classifying a data point, the geometric interval between the point and the hyperplane is larger, and the confidence is higher. Therefore, the optimal hyperplane of the structure can be maximized by several intervals to achieve the optimal solution of the classification. The known geometric interval remains unchanged with respect to the ratios of ω and *b*, so the objective function of the maximum interval classifier can be defined as $\max\tilde{\gamma }$, while satisfying $Y_{i}(\omega^{T}X_{i}+b)={\hat{\gamma }}_{i}\geq \hat{\gamma },\;i=1,\ldots ,n$, the function interval is $\hat{\gamma }=1$, then the objective function is:

(9) $$\max\frac{1}{\left\|\omega \right\|},\;(\omega^{T}X_{i}+b)={\hat{\gamma }}_{i}\geq 1,\;i=1,\ldots ,n.$$

The optimal hyperplane is found according to the objective function to complete the establishment of the classifier.

The confidence represents the geometric distance between the test image vector and the edge of an optimal hyperplane of each SVM. Therefore, the confidence corresponding to each SVM needs to be normalized to be compared. In this work, the confidence level is normalized in the range [0, 1] to facilitate the comparisons. The set *S_d_* is the set of corresponding terrain confidence sets for the test image from SVM; *D_i_* is the confidence degree of the *i* class terrain for the test image; *S_D_* is the normalized confidence set after the normalization; and *D_i_* is the confidence degree after the normalization. The normalization method for this topic is:

(10) $$S_{D}=\left\{D_{i}|\;D_{i}=\frac{\left|d_{i}\right|}{\sum_{i=1}^{6}\left|d_{i}\right|},\;i=1,2,\ldots ,6\right\}.$$

**Figure 4:**
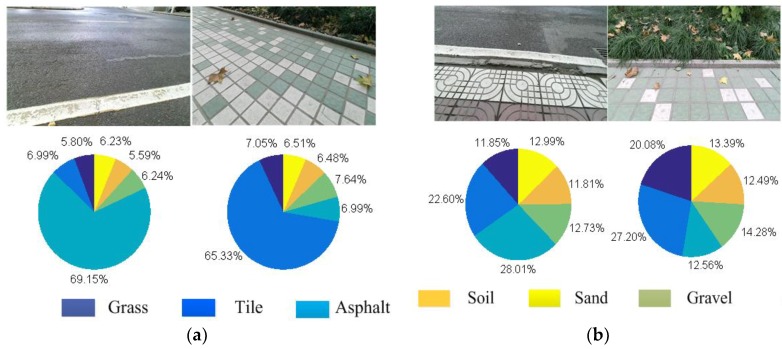
The membership pie charts after one recognition: (**a**) membership pie chart for a single terrain; and (**b**) membership pie chart for a complex terrain.

After normalization, the pie charts of confidence can clearly show the membership pie charts of various terrains corresponding to the terrain image. The set of membership pie charts for all terrain types after a single identification are presented in Figure 4.

**Figure 5:**
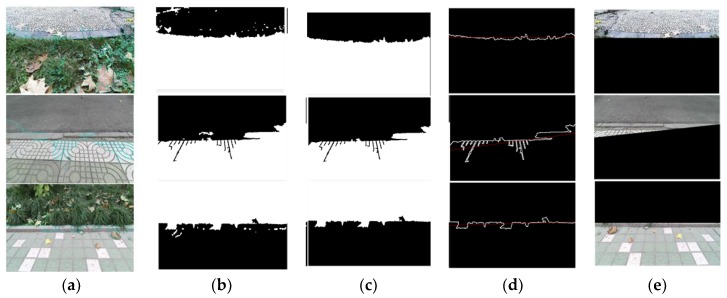
The segmentation result of the mixed-terrain images: (**a**) segmentation results of the SLIC algorithm; (**b**) maximum super-pixel extraction; (**c**) filtering out of smaller areas; (**d**) finding the boundary and fitting the line; and (**e**) finished segmentation.

In Figure 4, if the maximum proportion of a terrain is greater than the threshold (which is 30% in our work), and if it is higher (10%) than the second highest proportion, the overall terrain can be regarded as a single terrain and it can be accurately identified by SVM. However, in the mixed terrain, it is difficult to determine the category from the specific proportion in the pie chart. It should be noted that mixed terrain usually contains different terrain interactions. The traditional method is not practical because only one tag will be labeled. As shown in Figure 4b, not only is it difficult to accurately identify terrain type from the image, but also a single recognition result does not have any practical significance. Therefore, The SLIC segmentation algorithm is applied to the mixed-terrain region. In the superpixel region, the most pixels are selected as a target region, and the boundary pixels of the pixel coordinates of the curve fitting are extracted as a terrain boundary in the complex terrain, and the color image is segmented and filled. The processed color images are then sorted again by the terrain classifier to provide accurate identification of multiple areas of the complex terrain. Finally, all terrain types can be accurately predicted to ensure good performance of the robot. The terrain boundary segmentation results are shown in Figure 5. 

### 3.3. SLIC-SegNet Terrain Classification

Convolutional neural networks (CNNs) [36] have become a research hotspot in the image processing field. They have a weight-sharing structure similar to biological neural networks, which reduces the complexity of a network model, and a number of weights, and alleviates the overfitting problem of the model. The image can be directly used as a network input to avoid the complicated feature extraction and data reconstruction of the traditional recognition algorithms; in addition, a CNN structure can better adapt to the image structure. SegNet is a deep learning network proposed by Cambridge to solve the semantic segmentation of autopilot or intelligent robot images based on the Caffe framework. We perform training and testing on the original SegNet architecture, collect terrain images of the robot’s walking environment, and input images that need to be segmented. Then, we perform the convolution operations to extract the high-dimensional image features and make the images smaller through sampling and pooling. After deconvolution and downsampling, the features of the image classification are reconstructed. Finally, the maximum value of different classifications is output by the Softmax layer, and the segmentation result with a semantic filling is obtained.

In SegNet, the parameter solution mainly includes: the residuals of the convolution layer and the subsampling layer, and the corresponding weight parameters and derivatives of the offset parameters. In the convolutional layer, the feature map of the upper layer is convoluted with the learnable convolution kernel, and then output feature maps are obtained through an activation function.

(11) $$x_{j}^{l}=f(\sum_{i\in M_{j},}x_{i}^{l-1}\times k_{ij}^{l}+b_{j}^{l}),$$

where *M_j_* represents the set of selected input maps and the convolution is a “valid” boundary process. Each output map gives an additive deviation *b*. $k_{ij}^{l}$ stands for the convolution kernel. Then, the network learning is accelerated by rate through Batch Normalization [37], and the problem of gradient disappearance and gradient explosion is solved. For a given map, you can find its sensitivity map. The gradient of the bias basis is quickly calculated by summing all the nodes in the sensitivity map in layer *l*, and the gradient of the convolution kernel weight can be calculated by the BP algorithm. For a given weight, we need to find a gradient for all the connections that are related to the weight (the weight-shared join) and then sum these gradients. In the subsampling layer, there are *N* output maps for *N* input maps, but each output map becomes smaller. Where *down*( ) represents the downsampling function. Each output map has a multiplicative bias *β* and an additive bias *b* corresponding to it. Then, we can calculate the additive base *b* and the multiplicative base *β* gradient.

(12) $$x_{j}^{l}=f(\beta_{j}^{l}down(x_{j}^{l-1})+b_{j}^{l}).$$

The sensitivity of the fully connected layer *l* can be calculated by the following formula:

(13) $$\delta^{l}={\left(\omega^{l+1}\right)}^{T}\delta^{l+1}\circ f^{\prime }(u^{l}),$$

*f*’(*u^l^*’)stands for the derivative value of the activation function *f* of the current layer neuron node with input *u*. The partial derivative of the total error to the offset term is as follows:

(14) $$\frac{\partial E}{\partial b^{l}}=\frac{\partial E}{\partial u^{l}}\frac{\partial u^{l}}{\partial b^{l}}=\delta^{l}.$$

Next, each neuron can be updated with its sensitivity using weights. For a given fully connected layer *l*, the weight update direction can be represented by the inner product of input *x^l^*^−1^ and sensitivity *δ^l^* of the layer:

(15) $$\frac{\partial E}{\partial \omega^{l}}=x^{l-1}{\left(\delta^{l}\right)}^{T}.$$

**Figure 6:**
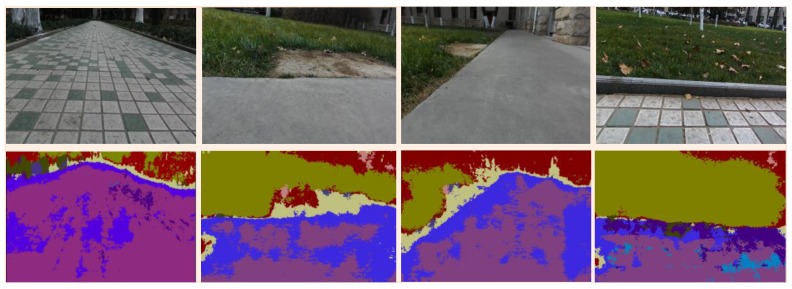
SegNet segmentation result. Color images and SegNet processing results of four different terrains.

The SegNet has made a very excellent work in the field. However, some problems are still left in application to legged robot. As shown in Figure 6, in the image after SegNet segmentation, the type of the object can be accurately identified, and semantic filling on various types can be performed. Compared with SVM classification, SegNet is more suitable for complex terrains, while the classification process is simple and accurate. However, in Figure 6, the boundary after SegNet segmentation is blurred, and there are some label-tagging errors. In traditional CNNs, Rectified Linear Unit (ReLU) layer is usually used after the full connection, combined with the bias to calculate the output of the weight. However, it is found in SegNet that more active layers will lead to better image semantic segmentation results [30]. Although the addition of feature activation can improve the accuracy of recognition and the clarity of the boundary to a certain extent, accurate and perfect classification results rely on computer hardware and cannot be obtained easily. For the legged robot, we only need to classify the terrain to ensure robot adaptability to the environment and its ability to choose the area independently. Therefore, here, the segmentation results are improved using SLIC. 

**Figure 7:**
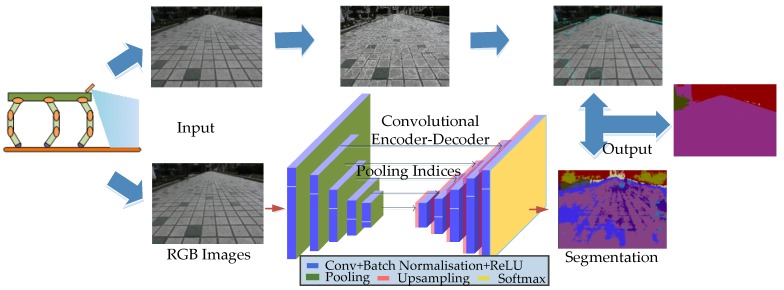
The flow chart of the SLIC-SegNet algorithm.

In this work, the SegNet and SLIC superpixel segmentation methods are combined to solve the problem of boundary blurring. The specific algorithm flow is as follows and shown in Figure 7. An input image is segmented, the terrain classification results with semantic filling are obtained by the SegNet terrain classifier, and superpixel segmentation is conducted on the image to be segmented. The image obtained by the former has a clear division of different terrains, each pixel of the image is assigned with the appropriate color label of the terrain, and its position is determined. The latter makes clear division of different types of terrain boundary in the image, and each kind of terrain takes the same marking symbol at the corresponding pixels. Then, each pixel in the SegNet terrain classification result corresponds to a pixel of the SLIC classification results. Each pixel in the SegNet result contains the RGB color information. The SLIC segmentation result is marked for each pixel point and the same terrain has the same marking symbol. First, the pixels corresponding to the same type of topographic marker in the SLIC are found, and then, the color value of the corresponding pixel in the SegNet result is assigned to the SLIC segmentation result to obtain a semantically segmented image with clear boundaries and semantic filling. The pseudo code of this algorithm is shown in Algorithm 1.

**Algorithm 1.** Pseudo code of SLIC-SegNet algorithm.Preparatory work for SegNet module [30]:  a: Convolution operation, get the feature value *x*;  b: Batch Normalizing Transform;  c: Training a Batch-Normalized Network; Result: the trained SegNet module.Initialize: *k* = {1 … *K*}, *i* = {1 … 480}, *j* = {1 … 360}, *n* = {1 … *N*}, *m* = { 1… *M*}. B_i_(*i*−1 ,…, *n*) represents the set of pixels in each sorted area, the RGB components of each pixel are denoted as *I_Bm_*(*x, y*); *I_An_*(*x, y*) stand for the pixels in $A_{n}^{k}$, *Mode* represents the component value of the most frequently occurring RGB components of all pixels in $A_{n}^{k}$, recorded as *I_Mode_* Repeat 1.  Collect *k_th_* image: *I_k_*(*x_i_, y_j_*)   For *k* = 1…*K* do 2.    Run the SLIC module:        Assign the best matching pixels;       Compute new cluster centers and residual error E;       until E ≤ threshold;       Enforce connectivity;       Output the each pixel block $A_{n}^{k}$ ∈ {*A*_1_, *A*_2_ … *A_N_*|$\sum_{n=1}^{N}A_{n}^{k}=I_{k}$}; where *A_n_* = *n* each pixel *I_An_(x, y)* ∈ $A_{n}^{k}$ 3.    Run the trained SegNet module:       Activate feature value;       Deconvolution, get the feature value *X^n^*;       Find the maximum probability of each pixel in all categories.       Output the label set $B_{m}^{k}$ ∈ {*B*_1_, *B*_2_ … *B*_M_|$\sum_{m=1}^{M}B_{m}^{k}=B_{k}$}, *I_Bm_*(*x, y*) ∈ $B_{m}^{k}$ 4.     Match $A^{k}\;and\;B^{k}$, define set $C_{n}^{k}$ = $A_{n}^{k}$        For *i* = 1 … *n*          find each *x_An_*, *y_An_* of *I_Cn_*          $I_{n}^{Mode}$ = *Mode(I_B_*(*x*_An_, *y*_An_));          For each (*x*_An_, *y*_An_) ∈ $A_{n}^{k}$            Assignthe *Mode* of the RGB component to *I_Cn_*(*x*_An_, *y*_An_):            *I_Cn_*(*x*_An_, *y*_An_) = $I_{n}^{Mode}$          End for         End for         Output $C_{n}^{k}$ ∈ {*C*_1_, *C*_2_ … *C_N_*|$\sum_{n=1}^{N}C_{n}^{k}=C_{k}$}, *I_Cn_*(*x, y*) ∈ $C_{n}^{k}$ 5.  Gait selection and run the Robot;    End for Until the Robot switched off.

The results of this work will provide clear boundaries and semantic segmentation results. At the same time, the segmentation results are optimized. To meet the requirements of the robot movement, it is necessary to provide the feasible terrain information quickly and accurately, and the terrain boundary provides a powerful basis to ensure the stability of the robot and make the gait adjustment in time. However, the boundary information of the SegNet classification results is relatively vague, and when the environmental information is more complex, or the terrain features of test and training samples are very different, the accuracy of segmentation results will be different. For robots, we need to determine the feasible area and the terrain boundary. Therefore, the SLIC superpixel segmentation results with clear boundary information are fused with the SegNet results. It can accurately capture the location and properties of the passable area. The SLIC-SegNet method makes an adaptive adjustment to the different terrains, and provides the basis for gait transformation and path planning.

## 4. Experiment

A series of experiments were conducted to verify the proposed methods. Both SLIC-SVM method and SLIC-SegNet method in single terrain and mixed terrain were tested, analyzed, and finally compared. 

### 4.1. SLIC-SVM Experiments

In the SLIC-SVM experiments, 30 images of mixed terrain in the campus including six different geological conditions, asphalt, grassland, tile, soil, gravel and sand, were captured. The images were collected on a sunny day with good light intensity. The camera was fixed on the robot front, and camera tilt was 40°. The test images were captured by the Kinect camera mounted on a hexapod robot walking on different terrains. It took one shot per second and the whole processing time of one image was about 0.2 s. In the tests, after the image segmentation, some output tags were not matched with the actual terrain types and some error tags are shown in Table 1.

**Figure 8:**
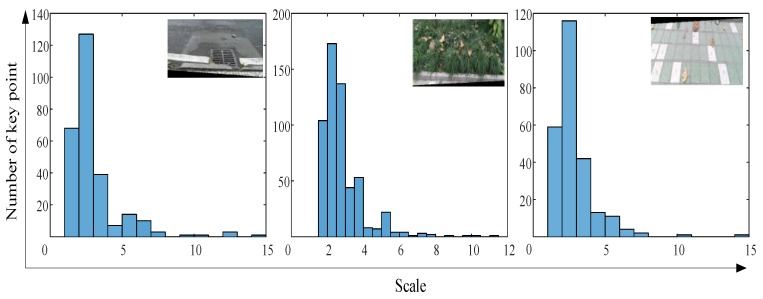
The number of feature points in segmented images.

**Figure 9:**
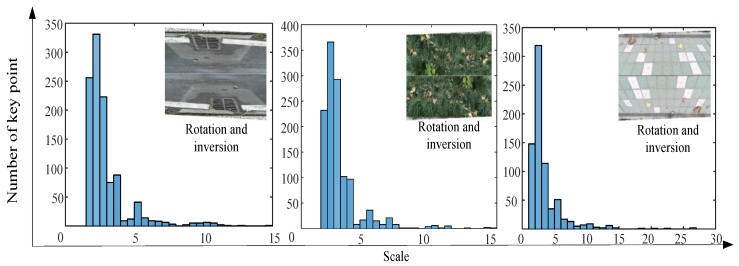
The number of feature points in spliced images.

To explain the mismatch, we extracted the number of SURF feature descriptor from the segmented images, as shown in Figure 8. Obviously, the feature points of the segmented image were not sufficient. Therefore, because of using the segmented image for terrain recognition, the accuracy of the output terrain label was reduced. To improve recognition accuracy, the image filling method was adopted to process the divided image to enhance the terrain features. The segmented color image contained only the pixels of the original color image collected by the Kinect camera, and the other part of the blank pixels was filled by copying the divided image. In the test, the rotation inversion operation was used for image filling. The number of feature points in Figure 8 and Figure 9 indicate that the proposed method can enhance the local features of the segmented image. The classification results of the stitched images using the proposed methods are shown in Table 1. Using the image filling method (rotation reversal), the error of the first classification round can be corrected. Obviously, the confidence score of the terrain type increased after the image was filled. In contrast, the confidence score of the wrong terrain type was reduced. The results are shown in Table 2. This means that the proposed method can effectively enlarge the image features of the classifier.

**Figure 10:**
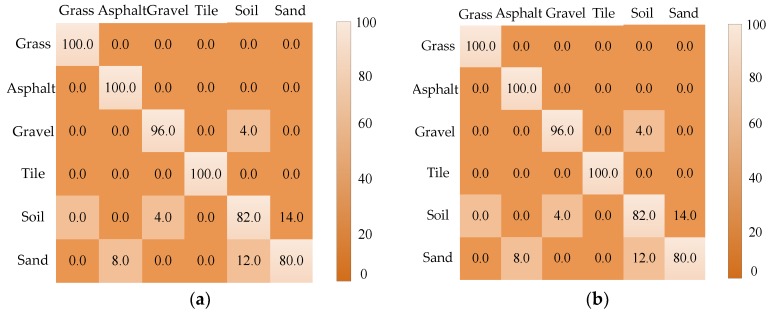
(**a**) Classification results of a single terrain; and (**b**) the recognition rate of the mixed terrain.

In the experiment, the six-legged robot walked on six terrain types without obstacles. The terrain image was collected by a Kinect camera mounted on the robot top. The tilt angle of the Kinect sensor was 40°. The recognition rate after 50 tests is shown in Figure 10, where it can be seen that the recognition accuracy of the grass, asphalt and floor tiles reached 100%, and the recognition accuracy of the other three terrain types was above 80%. The main factors affecting the classification result were the surface texture features of each terrain type and the number of extracted SURF feature descriptors. The recognition rates of soil and sand were the lowest because sand and soil were similar in surface texture and color. Terrain images were collected at different times and weather. The high reflectivity of sand under illumination resulted in fewer characteristic points. Therefore, the recognition accuracy was low. In general, the average recognition accuracy was higher than 80%.

### 4.2. SLIC-SegNet Experiments

The SegNet achieved an overall smooth segmentation of the maximum degree of association with all environments on the images. Even though there were trees, roads, and buildings on the image, accurate recognition of objects and segmentation of appearance surfaces can be achieved. However, the average accuracy of categories and the boundary division effect of categories are not ideal. Experimental results show that, when training samples were few and training time was short, the image segmentation results were poor, the accuracy was low, and the boundary information was more blurred. At the same time, the experimental results also validated the influence of the RGB as an input on the shape and texture of the object in the recognition process to reasonably and accurately implement the image segmentation process. The experimental results show that, when a certain training level was reached, the accuracy of the segmentation result became better. In this work, the Caffe 8.0 framework built on Ubuntu 16.04 was used. The Kinect camera was fixed on the robot top and used to capture images. The robot height was 40 cm, the tilt angle of the camera was 40°, and the pixel size was 480 × 360. The training sample used the dataset in reference [30], with a sample size of 367 and a training frequency of 40,000. The whole processing time of one image is about 0.6 s. Finally, accurate SegNet segmentation results were achieved.

The determination of boundary information and boundary division of superpixel segmentation denoted an important determinant of the final segmentation results, and the superpixel segmentation contained three important parameters: number of desired superpixels, weighting factor between color and spatial differences, regions morphologically smaller than this were merged with adjacent regions. The experimental results showed the influence of the superpixel segmentation parameters on the SLIC-SegNet segmentation results for the irregular, mixed images containing the sidewalks, buildings and the background information of the trees, and the most suitable parameters of superpixel segmentation were determined through experiments.

**Figure 11:**
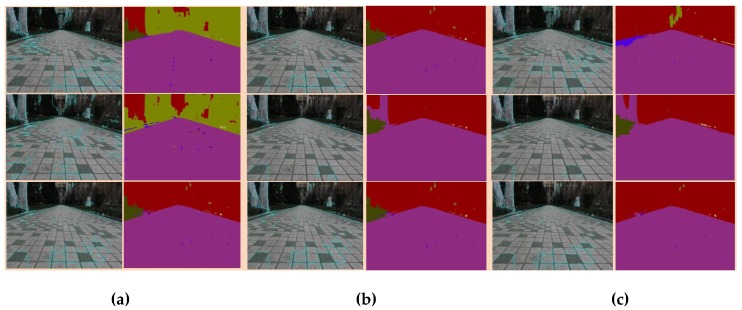
The effect of SLIC parameters. (**a**),(**b**),(**c**) is a three-group SLIC parameter selection experiment, including color images and SLIC segmentation results. From left to right, top to bottom, the parameter values are in order: (10, 20, 1), (45, 20, 1), and (100, 20, 1); (45, 10, 1), (45, 30, 1), and (45, 40, 1); and (45, 20, 1.2), (45, 20, 1.3), and (45, 20, 1.5). Red color represents the building, purple color represents the sidewalk, and light green color represents the trees.

The classification and recognition of terrain by a robot should enable the robot to judge the unknown environment and provide timely gait transition, so it is reasonable to mark the mixed background of the building and trees as the building part. The superpixel segmentation contained three important parameters: number of desired superpixels, weighting factor between color and spatial differences. The number of superpixels defines size of each superpixel in the segmentation result. When weighting factor between color and spatial differences was larger, the boundary became blurred. To maximize the color distance and balance the color similarity and spatial similarity, the weight factor should be set. The superpixel parameters selected in this paper are the parameters applicable to this experiment obtained through many experiments according to the above principles. In Figure 11, the segmentation result contained the overfitting, the shape of the segmentation was irregular, the neighborhood relationship was difficult to maintain, the number of divisions was large, and so the number of superpixels was selected to be 45. When weighting factor between color and spatial differences was larger, the boundary became blurred. To maximize the color distance and balance the color similarity and spatial similarity, the weight factor was set to 20. For instance, at (10, 20, 1) and (45, 10, 1), where the numbers in the brackets denote the values of the above-mentioned three parameters, the terrain recognition mistakenly identified trees as buildings and mixed background of buildings and trees as trees, and at (45, 30, 1) and (45, 40, 1), when the weight factor of color and space difference was large, the tree was taken as a sidewalk. The merging parameter of regions morphologically smaller than this are merged with adjacent regions is larger, the boundary of the segmentation was unclear. At (45, 20, 1.5), the grassland and buildings were all recognized as buildings. Therefore, we selected the set (45, 20, 1) to identify the sidewalk and the grassland correctly, and for the mixed terrain containing the trees and buildings as an infeasible area, the marking process for robot was in line with the requirements.

### 4.3. Comparison of SLIC-SVM and SLIC-SegNet 

To compare the SLIC-SVM and SLIC-SegNet, the experiment was conducted. The experiment was performed and each image was recognized by two methods, respectively. The superpixel segmentation parameters were: (45, 20, 1). A Caffe 8.0 framework built on Ubuntu 16.04 was used. The training sample used the dataset in Ref. [30] with a sample size of 367 and a training frequency of 40,000. The test image is a color image captured by the Kinect camera in real time. The superpixel parameter is (45, 20, 1).

**Figure 12:**
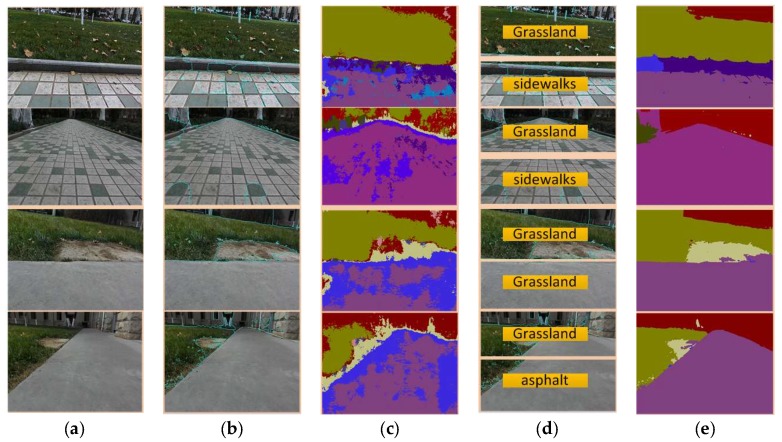
The terrain classification results of the SLIC-SVM and SLIC-SegNet: (**a**) images to be processed; (**b**) SLIC results; (**c**) SegNet results; (**d**) SLIC-SVM results; and (**e**) SLIC-SegNet results.

It can be seen in Figure 12 that, in the complex irregular mixed terrain environment, the SLIC-SVM terrain classification method cannot perform correct image segmentation, resulting in terrain tag recognition errors. For example, a mixed terrain with irregular boundaries of various terrain types including grassland, land, and sidewalks is mistakenly judged as a single terrain: grassland. SegNet image semantic segmentation, although roughly meeting the requirements of complex mixed terrain classification, as can be seen from the experiment in Figure 12c, the boundary information of the mixed terrain is very blurred, and will lead the robot to make the incorrect gait transition at the terrain boundary, which may cause the robot to be unstable. The SLIC-SegNet terrain classification method solves the classification problem that the SLIC-SVM cannot identify the mixed terrain with irregular boundaries. On the other hand, it optimizes the SegNet terrain classification effect and obtains the terrain classification result with clear boundary and high accuracy. It provides a strong basis for the gait transition and path planning of the robot to meet the stability requirements during the process.

After the comparison of the SLIC-SVM and SLIC-SegNet, the analysis of the results was conducted to prove the superiority of the SLIC-SegNet terrain classification method, and the following conclusions were made:

(1) In contrast to the terrain classification method based on the SLIC-SVM, the convolutional neural network terrain classification method based on the superpixel segmentation belongs to the single-input multi-output model. Using a single image of a mixed terrain multiple terrain recognition and marking processes can be achieved simultaneously. However, for the SLIC-SVM of single-input single-output model, it is necessary to divide different terrains first, and then to identify them separately.
(2) In the mixed terrain classification by the SLIC-SVM terrain classification method, different terrains need to be segmented, and then feature points are extracted to recognize the terrains. However, the reduction of the number of feature points after image segmentation inevitably leads to the low terrain recognition rate. The SLIC-SegNet can process the input image without segmentation ensuring the requirement for pixels and feature points of the segmentation process, and can identify a variety of mixed terrain accurately and quickly.
(3) The SLIC-SVM can divide only the mixed terrain with the regular terrain features. The mixed terrain with irregular terrain features cannot be segmented, and the terrain cannot be identified accurately. The SLIC-SegNet terrain classification method can accurately identify each terrain type, even the irregular mixed terrains.

## 5. Discussion

The terrain recognition is always used for gait transition and path planning of robots in the process of moving. Therefore, terrain classification results with clear boundaries and semantic filling are needed. The robot is enabled to judge the terrain timely and perform appropriate gait transformation and path planning. Hence, the environmental adaptability and self-selection capabilities of the robot during the movement can be improved. The SLIC segmentation technology is used to complete the terrain segmentation process, and the improved terrain identification methods are combined with the SVM and SegNet terrain recognition method to obtain the terrain classification results with clear boundaries and accurate terrain labels.

Actually, we proposed a synthetic classification method to obtain both advantages of segmentation methods and classification methods. Most terrain classifications are mainly extracting different types of terrain features and establishing classifiers. Terrain classifiers in robot are commonly based on the SVM [8] and neural networks [36,37,38]. Although these terrain classification methods can complete terrain recognition, the boundary information is not accurately determined, so a robot cannot adjust the gait timely and accurately. Even the popular deep learning method [39,40] will not show the clear boundary information. Thus, segmentation method cannot be avoided to be used.

Segmentation methods such as SLIC [16], watershed [19,20] and level-set [21,22] are commonly used. The watershed segmentation technique is a region-based segmentation method to obtain continuous and closed target boundaries with fast processing speed. However, it is easy to produce over-segmentation, which is very sensitive to noise and fine texture. The level-set technology has the characteristics of compactness and high edge matching, but its image segmentation speed is slow, and it is easy to cause boundary leakage phenomenon, which makes the segmentation result less accurate. The SLIC processing speed is fast, the memory is smaller, the edge is more consistent, and the segmentation performance is good [41,42].

The robots in this paper are in a complex field environment, so it is more appropriate to use visual features to complete the convolutional neural network architecture. The SLIC-SegNet terrain classification method proposed in this paper uses the advantage of SLIC in image segmentation and CNN for visual feature extraction in image classification, and solves the problem that the CNN cannot clearly divide the boundary in the terrain classification process. The synthetic terrain classification methods are more suitable for field autonomous navigation robots. 

## 6. Conclusions

To provide better path planning and gait transformation of the hexapod robots, two superpixel segmentation based synthetic classification methods are proposed. The SLIC is fused with both the SVM and the Segnet. Firstly, the SLIC is used to divide the mixed terrain and capture the terrains boundary; then, the image is subjected to image segmentation and the SVM terrain classifier based on the SURF method is used for terrain classification. In this way, the problem that the SVM can only recognize a single terrain is solved. In the SLIC-SegNet method, the terrain classification and semantic filling are obtained by the SegNet. Then, in the segmentation results obtained by the SLIC superpixel segmentation, an area corresponding to the SegNet classification result is found. Thus, the semantic filling results of the SegNet classification are assigned to the results of the SLIC terrain segmentation to get a clear and semantically filled terrain classification. The experimental results proved that the both methods are effective. The presented results have an important guidance for the gait transformation and locomotion control of the legged robot.

The theoretical contributions and novelty of this work can be summarized as follows:

1. The SLIC-SVM is proposed to solve the problem that the SVM can only output a single terrain label and fail to identify the mixed terrain. The presented method can not only recognize a variety of mixed terrains but also provide the clear terrain boundary for gait transformation and stability of multi-legged robot.
2. The SLIC-SegNet single-input multi-output terrain classification model is derived to improve the applicability of the terrain classifier. Since terrain classification results of high quality for legged robot are hard to gain, the SLIC-SegNet obtains the satisfied information without too much effort.
3. Both superpixel segmentation based synthetic classification methods can supply reliable mixed terrain classification result with clear boundary information and will put the terrain depending gait selection and path planning of the multi-legged robots into practice.

Therefore, the proposed terrain classification methods based on the SLIC supplies the robot with real and reliable terrain information enabling the robot to adjust its gait timely and stably during the movement. Consequently, this provides the basis for autonomous gait selection and path planning, which further makes a multi-legged robot more intelligent and autonomous in an unknown environment. To improve the application of terrain classification in the field of autonomous navigation robots, in future research, we will focus on terrain information, such as geometrical shape, characteristics of terrains and coupling characteristics of environment and robot, to improve the behavior selection and fast transition of robot gait. Better environmental cognition and understanding will greatly contribute to the outdoor walking of robot.

## Figures and Tables

**Table 1:** Segmented terrain classification results.

**Images**	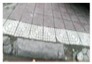	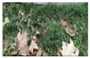	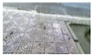	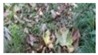	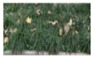
**Actual terrain**	Tile	Grass	Tile	Grass	Grass
**Output label**	Asphalt	Tile	Asphalt	Asphalt	Soil

**Table 2:** Confidence scores after splicing recognition.

Actual Terrain	Tile		Grass		Tile		Grass		Grass	
Output Label	Asphalt		Tile		Asphalt		Asphalt		Soil	
R-I	Tile		Grass		Tile		Grass		Grass	
Scores	Before	After	Before	After	Before	After	Before	After	Before	After
Sand	12.21	12.78	14.55	11.94	12.53	12.18	11.19	10.41	12.95	7.59
Grass	10.40	13.33	14.56	29.30	12.88	14.92	24.59	39.52	16.71	59.12
Asphalt	33.39	14.29	19.72	13.47	25.05	18.32	26.70	11.16	18.17	7.96
Gravel	12.73	13.41	17.71	20.01	12.27	12.79	12.66	15.61	15.85	8.17
Tile	20.38	35.05	19.95	12.96	24.86	29.37	12.27	11.41	16.12	8.16
Soil	10.89	11.15	13.51	12.33	12.42	12.42	12.59	11.89	20.20	9.30

## References

[1] Zhu Y.G., Jin B. (2016). Trajectory Correction and Locomotion Analysis of a Hexapod Walking Robot with Semi-Round Rigid Feet. Sensors.

[2] Ji A.H., Dai Z.D. (2005). Research Development of Bio-inspired Robotics. Robot.

[3] Abbaspour R. Design and implementation of multi-sensor based autonomous minesweeping robot. Proceedings of the Ultra Modern Telecommunications & Control Systems & Workshops International Congre.

[4] Caltabiano D., Muscato G., Russo F. Localization and self-calibration of a robot for volcano exploration. Proceedings of the IEEE International Conference on Robotics and Automation(ICRA).

[5] Zhao S.D., Yuh J.K. (2005). Experimental Study on Advanced Underwater Robot Control. IEEE Trans. Robot..

[6] Cui Y., Gejima Y., Kobayashi T. (2013). Study on Cartesian-Type Strawberry-Harvesting Robot. Sens. Lett..

[7] Semler L., Furst J. Wavelet-Based Texture Classification of Tissues in Computed Tomography. Proceedings of the 18th IEEE Symposium on Computer-Based Medical Systems (CBMS'05).

[8] Paschos G. (2001). Perceptually uniform color spaces for color texture analysis: An empirical evaluation. IEEE Trans. Image Process..

[9] Khan Y., Komma P., Bohlamnn K. Grid-based visual terrain classification for outdoor robot using local features. Proceedings of the IEEE Conference on IEEE Symposium on Computational Intelligence in Vehicles and Transportation Systems.

[10] Zenker S., Aksoy E.E., Goldschmidt D. Visual Terrain Classification for Selecting Energy Efficient Gaits of a Hexapod Robot. Proceedings of the 2013 IEEE/ASME International Conference on Advanced Intelligent Mechatronics.

[11] Filitchkin P., Byl K. Feature-based terrain classification for LittleDog. Proceedings of the IEEE/RSJ International Conference on Intelligent Robot & Systems.

[12] Bao S., Chung A.C.S. (2018). Multi-scale structured CNN with label consistency for brain MR image segmentation. Comput. Methods Biomech. Biomed. Eng.: Imaging Vis..

[13] Rothrock B., Kennedy R., Cunningham C., Papon J., Heverly M., Ono M. Spoc: Deep learning-based terrain classification for Mars rover missions. Proceedings of the AIAA SPACE 2016.

[14] Levinshtein A., Stere A., Kutulakos K.N., Fleet D.J., Dickinson S.J., Siddiqi K. (2009). Fast superpixels using geometric flows. IEEE Trans. Pattern Anal. Mach. Mach..

[15] Stutz D., Hermans A., Leibe B. (2018). Superpixels: An evaluation of the state-of-the-art. Comput. Vis. Image Underst..

[16] Ren X., Malik J. Learning a Classification Model for Segmentation. Proceedings of the IEEE International Conference on Computer Vision.

[17] Song X., Zhou L., Li Z. (2015). Review on superpixel methods in image segmentation. J. Image Graph..

[18] Nguyen B.P., Heemskerk H., So P.T.C., Tucker-Kellogg L. (2016). Superpixel-based segmentation of muscle fibers in multi-channel microscopy. BMC Syst. Biol..

[19] Chen X., Nguyen B.P., Chui C.K., Ong S.H. Automated brain tumor segmentation using kernel dictionary learning and superpixel-level features. Proceedings of the 2016 IEEE International Conference on Systems, Man, and Cybernetics (SMC).

[20] Marcin C. (2015). Automated coronal hole segmentation from Solar EUV Images using the watershed transform. J. Vis. Commun. Image Represent..

[21] Cousty J., Bertrand G., Najman L., Couprie M. (2010). Watershed cuts: Thinnings, shortest path forests, and topological watershed. IEEE Trans. Pattern Anal. Mach. Intell..

[22] Zhang K., Zhang L., Lam K.M., Zhang D. (2016). A level set approach to image segmentation with intensity inhomogeneity. IEEE Trans. Cybern..

[23] Min H., Jia W., Wang X.F., Zhao Y., Hu R.X., Luo Y.T. (2015). An intensity-texture model based level set method for image segmentatio. Pattern Recognit..

[24] Holder C.J., Breckon T.P. (2016). From On-Road to Off: Transfer Learning within a Deep Convolutional Neural Network for Segmentation and Classification of Off-Road Scenes. European Conference on Computer Vision, Proceedings of the Computer Vision—ECCV 2016 Workshops, Amsterdam, the Netherlands, 8–10 and 15–16 October 2016.

[25] Ordonez C. (2013). Terrain identification for RHex-type robots. Unmanned Syst. Technol. XV.

[26] Lee S.Y., Kwak D.M. A terrain classification method for UGV autonomous navigation based on SRUF. Proceedings of the International Conference on Ubiquitous Robots & Ambient Intelligence.

[27] Dallaire P. Learning Terrain Types with the Pitman-Yor Process Mixtures of Gaussians for a Legged Robot. Proceedings of the Intelligent Robots and Systems (IROS).

[28] Manduchi R., Castano A., Talukder A. (2005). Obstacle Detection and Terrain Classification for Autonomous Off-Road Navigation. Auton. Robot..

[29] Greiffenhagen M., Ramesh V., Comaniciu D., Niemann H. Statistical modeling and performance characterization of a real-time dual camera surveillance system. Proceedings of the IEEE Conference on Computer Vision and Pattern Recognition (CVPR).

[30] Badrinarayanan V., Handa A., Cipolla R. (2015). Segnet: A deep convolutional encoder-decoder architecture for robust semantic pixel-wise labelling. arXiv.

[31] Hoepflinger M.A., Remy C.D., Hutter M., Haag S., Siegwart R. Haptic Terrain Classification on Natural Terrains for Legged Robots. Proceedings of the International Conference on Climbing & Walking Robot.

[32] Hoffmann M., Štěpánová K., Reinstein M. (2014). The effect of motor action and different sensory modalities on terrain classification in a quadruped robot running with multiple gaits. Robot. Autonom. Syst..

[33] Zhu Y., Wu Y.S., Liu Q. (2018). A backward control based on σ -Hopf oscillator with decoupled parameters for smooth locomotion of bio-inspired legged robot. Robot. Autonom. Syst..

[34] Bay H., Ess A., Tuytelaars T., Gool V.L. (2008). Speeded-Up Robust Features (SURF). Comput. Vis. Image Underst..

[35] Comaniciu D., Meer P. (2002). Mean shift: A robust approach toward feature space analysis. IEEE Trans. Pattern Anal. Mach. Intell..

[36] Rekeczky C. (2002). CNN architectures for constrained diffusion based locally adaptive image processing. Int. J. Circuit Theory Appl..

[37] Loffe S., Szegedy C. (2015). Batch Normalization: Accelerating Deep Network Training by Reducing Internal Covariate Shift. arXiv.

[38] Achanta R., Shaji A., Smith K., Lucchi A., Fua P., SãSstrunk S. (2012). Slic superpixels compared to state-of-the-art superpixel methods. IEEE Trans. Pattern Anal. Mach. Intell..

[39] Gonzalez R., Iagnemma K. (2018). DeepTerramechanics: Terrain Classification and Slip Estimation for Ground Robots via Deep Learning. arXiv.

[40] Valada A., Spinello L., Burgard W. (2017). Deep Feature Learning for Acoustics-Based Terrain Classification.

[41] Maghsoudi O.H. Superpixels based marker tracking vs. hue thresholding in rodent biomechanics application. Proceedings of the 2017 51st Asilomar Conference on Signals, Systems, and Computers.

[42] Maghsoudi O.H., Vahedipour A., Robertson B., Spence A. (2018). Application of Superpixels to Segment Several Landmarks in Running Rodents. arXiv.

